# Mesh retraction correlates with vaginal pain and overactive bladder symptoms after anterior vaginal mesh repair: reply to comment by Jacquetin

**DOI:** 10.1007/s00192-014-2334-9

**Published:** 2014-02-25

**Authors:** A. Rogowski, W. Baranowski, P. Bienkowski

**Affiliations:** 1 Department of Gynecology and Oncological Gynecology, Military Institute of Medicine, Warsaw, Poland; 2 Department of Pharmacology, Institute of Psychiatry and Neurology, Warsaw, Poland

Dear Editor,

We appreciate Jacquetin’s interest in our paper [1] and his insightful comments on ultrasound evaluation of postoperative mesh retraction after vaginal mesh repair [2]. In agreement with our results [1], Jacquetin has found a significant correlation between mesh retraction and the severity of postoperative vaginal pain [2]. The latter observation is of crucial significance as the measures used to evaluate mesh retraction by Jacquetin and by our group were different. Thus, the relationship between mesh retraction and postoperative vaginal pain can be observed regardless of whether mesh thickness (combined with a description of deformations) [2] or mesh length [1] is used as a primary measure.

Different processes thought to stand behind mesh retraction (e.g., insufficient spreading, shrinkage, and folding) were addressed in the “Introduction” section of our paper [1]. There is no single, widely accepted method of ultrasound evaluation of mesh retraction. We measured the mesh length with the aid of introital/transvaginal two-dimensional ultrasound [1]. Images were orientated according to the international recommendations [3], and the inter-rater reliability of ultrasound measurements was very good. In line with the previous suggestions [4], Jacquetin mentioned the area evaluation as the “ideal” measure of mesh retraction. It is our feeling that even this measure may not reflect all possible postoperative deformations in mesh shape. One could consider some sort of 3D imaging as an ideal approach to the problem.

We believe that more prospective studies are needed to select the best method of postoperative assessment of mesh retraction offering an optimal balance between validity, reliability, and feasibility.
